# Molecular Targets and Mechanisms of *Hedyotis diffusa*-*Scutellaria barbata* Herb Pair for the Treatment of Colorectal Cancer Based on Network Pharmacology and Molecular Docking

**DOI:** 10.1155/2022/6186662

**Published:** 2022-06-06

**Authors:** Zhenpeng Yang, Shuai Lu, Huazhen Tang, Jinxiu Qu, Bing Wang, Yuying Wang, Guofeng Pan, Benqiang Rao

**Affiliations:** ^1^Department of Gastrointestinal Surgery, Beijing Shijitan Hospital, Capital Medical University, Beijing 100038, China; ^2^Key Laboratory of Cancer FSMP for State Market Regulation, Beijing 100038, China; ^3^Department of TCM, Beijing Shijitan Hospital, Capital Medical University, Beijing 100038, China

## Abstract

*Objective*: *Hedyotis diffusa*-*Scutellaria barbata* herb pair (HS) has therapeutic effects on a variety of cancers, and this study aims to systematically explore the multiple mechanisms of HS in the treatment of colorectal cancer (CRC). *Methods*. The active ingredients of HS were obtained from TCMSP, and the potential targets related to these ingredients were screened from the STITCH, SuperPred, and Swiss TargetPrediction databases. Targets associated with CRC were retrieved by Drugbank, TTD, DisGeNET, and GeneCards. We used a Venn diagram to screen the intersection targets and used Cytoscape to construct the herb-ingredient-target-disease network, and the core targets were selected. The Go analysis and KEGG pathway annotation were performed by R language software. We used PyMol and Autodock Vina to achieve molecular docking of core ingredients and targets. Results: A total of 33 active ingredients were obtained from the HS, and 762 CRC-related targets were reserved from the four databases. We got 170 intersection targets to construct the network and found that the four ingredients with the most targets were quercetin, luteolin, baicalein, and dinatin, which were the core ingredients. The PPI analysis showed that the core targets were STAT3, TP53, MAPK3, AKT1, JUN, EGFR, MYC, VEGFA, EGF, and CTNNB1. Molecular docking results showed that these core ingredients had good binding potential with core targets, especially the docking of each component with MAPK obtained the lowest binding energy. HS acts simultaneously on various signaling pathways related to CRC, including the PI3K-Akt signaling pathway, proteoglycans in cancer, and the MAPK signaling pathway. Conclusions: This study systematically analyzed the active ingredients, core targets, and central mechanisms of HS in the treatment of CRC. It reveals the role of HS targeting PI3K-Akt signaling and MAPK signaling pathways in the treatment of CRC. We hope that our research could bring a new perspective to the therapy of CRC and find new anticancer drugs.

## 1. Introduction

Colorectal cancer (CRC) is a common digestive tract tumor, which has a high incidence rate, a high mortality rate, rapid progress, and easy spread [1]. The incidence rate of CRC is increasing year by year, the age of onset is getting younger and younger, and the death caused by recurrence and metastasis is still a considerable challenge [2]. Modern pharmacological studies have shown that traditional Chinese medicine (TCM) is one of the comprehensive treatments for CRC in addition to surgery, chemotherapy, radiotherapy, immunotherapy, and targeted therapy, which can effectively inhibit the proliferation of cancer cells and improve the quality of life [3–5]. Compared with side effects of chemotherapy, such as myelosuppression, gastrointestinal reaction, liver function damage, and peripheral neuritis, TCM has the characteristics of long-term administration, fewer side effects, and less drug resistance [6, 7].

TCM believes that cancer toxin is the core of tumor pathogenesis. After cancer toxin is produced, it can induce pathological products such as phlegm turbidity, qi stagnation, and blood stasis [8]. Blood stasis is closely related to tumor and runs through the whole process of swelling. Therefore, clearing heat and detoxification, promoting blood circulation, and removing blood stasis play an essential role in tumor treatment [9]. *Hedyotis diffusa* belongs to the genus Hedyotis of Rubiaceae, and it is bitter, light, and cold in nature [10]. *Scutellaria barbata*, belonging to Labiatae, is sour in taste and cold in nature [11]. They have the functions of clearing away heat and detoxification, promoting blood circulation and removing blood stasis, anti-inflammatory and analgesic effects, and can be used as adjuvant treatment for colorectal cancer, breast cancer, bladder cancer, lung cancer, liver cancer, gastric cancer, ovarian cancer, and other malignant tumors [12, 13]. Their pharmacological effects include antitumor, anti-inflammatory, antioxidation, antiangiogenic, promoting cell apoptosis, and improving immune capacity [14, 15]. *Hedyotis diffusa* and *Scutellaria barbata* herb pair (HS) are widely used to treat cancer, and their combined effects are more than a single use of drugs. Studies have shown that HS can inhibit the proliferation of human breast cancer cells, and the combination of CTX treatment can significantly inhibit breast cancer model mice [16, 17]. In addition, it could also significantly inhibit the growth of H22 hepatoma xenografts in mice [18]. The mixture of ethanol extracts from HS can dramatically inhibit the growth of human colon cancer cell lines compared with the single drug alcohol extract, which indicates that the anticancer effect of the combination of the two drugs will be enhanced. However, these studies focus on a single target or a single pathway, which could not comprehensively and systematically explain the antitumor effect of HS [19].

**Figure 1:**
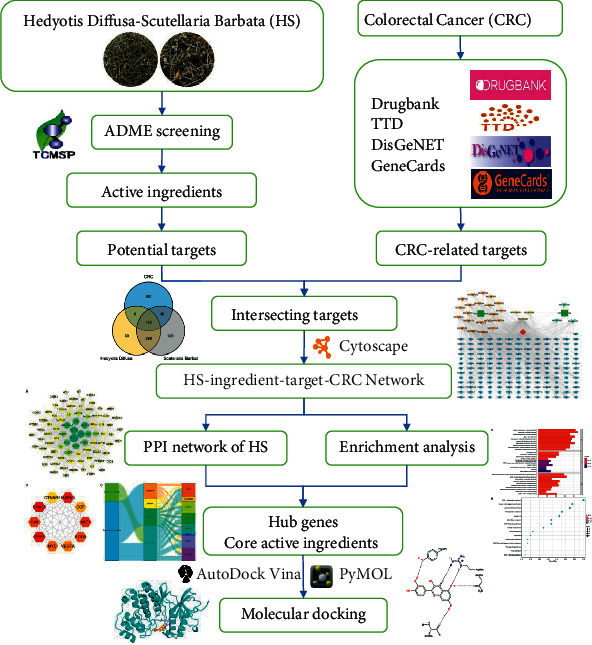
Flowchart of the study.

Network pharmacology emphasizes the combination of bioinformatics, system biology, and pharmacology, which not only explains the interaction between TCM and diseases but also conforms to the systematic and holistic view of TCM [20, 21]. Network pharmacology updates the “one target, one drug” model to the “multicomponent, multitarget” model and clarifies the complex interactions between genes, proteins, and metabolites related to diseases and drugs from the perspective of the network, which provides the possibility for us to systematically study the relationship between TCM and diseases [22, 23]. In this study, network pharmacology was used to analyze the active ingredients, potential targets, and main mechanisms of HS in the treatment of CRC, and to construct the herb-ingredient-target-disease network, so as to provide a reference for the study of the specific mechanism of the drug in the treatment of CRC. The flowchart of our analysis is shown in Figure 1.

## 2. Materials and Methods

### 2.1. Collection of Active Ingredients

With the common name of a single drug as a keyword, all chemical ingredients of the drug were retrieved by TCMSP [24] (https://tcmspw.com/tcmsp.php). This study used oral bioavailability (OB) and ingredient drug-likeness (DL) as screening conditions for active ingredients.

### 2.2. Prediction of Potential Targets of HS

We used PubChem [25] (https://pubchem.ncbi.nlm.nih.gov) to search and export the chemical structure data of active ingredients. Since the targets of ingredients without accurate structural information could not be predicted successfully, we decided to remove these ingredients after deleting the duplicate data. The active ingredients were predicted through the STITCH [26] (http://stitch.embl.de/), SuperPred [27] (http://prediction.charite.de/), and Swiss TargetPrediction [28] (http://www.swisstargetprediction.ch/) databases to obtain the corresponding known or predicted targets. The duplicate data had been eliminated, and only the human targets were retained.

The selected active ingredients were imported into the STITCH database for putative target prediction, and the targets with a confidence ≥0.7 were assumed as potential targets. The potential targets of drugs can also be obtained by inputting SMILES into the SuperPred database. The Canonical SMILES of the main active ingredients were uploaded to the Swiss TargetPrediction database, and the probability of each potential target was determined to be greater than or equal to 0.1. The retrieved targets were converted into standardized abbreviations by UniProt [29] (https://www.uniprot.org).

### 2.3. Collection of CRC-Related Targets

Targets associated with CRC were collected from Drugbank [30] (https://www.drugbank.ca), TTD [31] (http://db.idrblab.net/ttd/), DisGeNET [32] (http://www.disgenet.org/), and GeneCards [33] (https://www.genecards.org). Then the retrieval results of these databases were merged, and only one repeated target was reserved.

### 2.4. Intersection Targets and Network Construction

The targets of HS and CRC were intersected by R language version 4.1.1 and the common gene was identified as the intersection target of HS and CRC. The herbs, active ingredients, intersection targets, and diseases were introduced into Cytoscape 3.8.0 [34] to construct the herb-ingredient-target-disease network.

### 2.5. Protein-Protein Interaction (PPI) Analysis

The intersection targets related to HS and CRC were input into the STRING [35] (https://string-db.org/) platform for retrieval. Protein interaction data with high confidence (score >0.7) were selected and saved in a TSV format file. The information of node1, node2, and combined score in the file was imported into the software of Cytoscape to construct a PPI network, and the hub gene was screened by the cytohubba plug-in. The Sankey diagram revealed the relationship between herbs, core ingredients, and hub genes was drawn in R language version 4.1.1.

### 2.6. Enrichment Analysis

We applied the “clusterProfiler” package to these overlapping genes in order to perform GO enrichment analysis and KEGG analysis [36]. Both the bar plot and dot plot were drawn using the R language version 4.1.1.

### 2.7. Molecular Docking

We downloaded the SDF format 3D structure file of key ingredients from the PubChem database and converted it to PDB format through Open Babel. We also downloaded the 3D crystal structure of the hub genes through the PDB database (https://www.pdbus.org/), removed ions and water molecules through PyMol 2.4.0 [37], and saved it as a PDB file. Then we repaired the protein structure through the WHAT IF server website (https://swift.cmbi.umcn.nl/servers/html/model.html) and prepared the receptor file in Autodock Vina. Then, the molecular docking simulation was carried out by using Autodock Vina software and visualized by PyMol 2.4.0. The 3D and 2D diagrams of molecular docking models were displayed by PyMol 2.4.0 and PROTEINS PLUS (https://proteins.plus/), respectively.

## 3. Results

### 3.1. Screening of Active Ingredients

37 ingredients were found in *Hedyotis diffusa* and 94 in *Scutellaria barbata* from TCMSP database. OB ≥ 30% and DL ≥ 0.18 were selected as the screening conditions. There were 7 ingredients from *Hedyotis diffusa* and 29 from *Scutellaria barbata*. A total of 33 ingredients were screened. Among them, 3 ingredients were common ingredients of the two herbs, namely quercetin (MOL000098), beta-sitosterol (MOL000358), and stigmasterol (MOL000449). The basic information of active ingredients in HS is shown in Table 1.

### 3.2. Potential Targets of HS

The structures of these active ingredients were obtained in PubChem. We removed six ingredients named MOL001646, MOL000953, MOL005869, MOL012248, MOL012250, and MOL012270, which had no accurate structural information for predicting the target. The structures of the active ingredients were imported into STITCH, SuperPred, and Swiss Target Prediction databases for target prediction. A total of 490 potential targets of *Hedyotis diffusa* and 589 targets of *Scutellaria barbata* were obtained.

### 3.3. Collection of CRC-Related Targets

**Figure 2:**
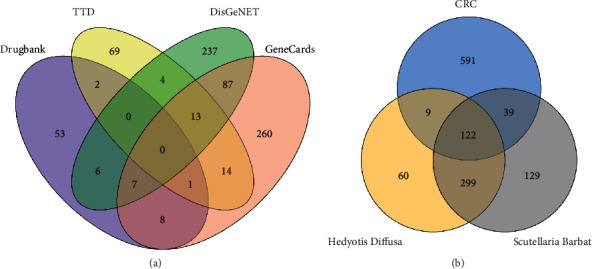
(a) Venn diagram showing the CRC-related targets among the four databases. (b) Venn diagram showing the intersecting targets.

77, 104, 353, and 390 CRC targets were obtained from four databases, including Drugbank, TTD, DisGeNET, and GeneCards, respectively, shown in the Venn diagram (Figure 2(a)). A total of 762 CRC-related targets were reserved from the four databases.

### 3.4. Construction of the Herb-Ingredient-Target-Disease Network

**Figure 3:**
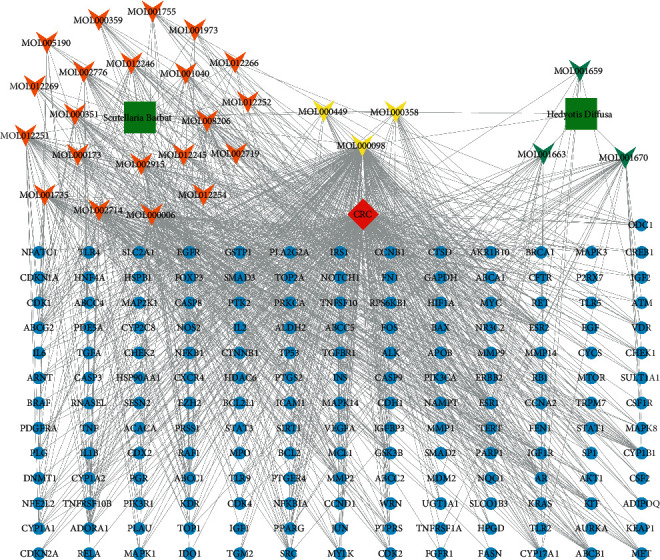
The herb-ingredient-target-disease network of HS against CRC. The green square represents the herb, the inverted triangles of different colors represent the ingredients of different herbs, the blue circle represents the target, the red diamond represents the disease.

We intersected the potential targets of ingredients and CRC-related targets, and a total of 170 intersecting targets were obtained (Figure 2(b)), among which 131 genes were shared by *Hedyotis diffusa* and CRC, and 161 genes were shared by *Scutellaria barbata* and CRC. Then, the two herbs, 27 active ingredients, 170 intersection targets, and CRC disease were imported into Cytoscape 3.8.0 to construct the herb-ingredient-target-disease network (Figure 3). In the network, the more edges a node connects with other nodes, the higher its degree value. The node with a high degree value may be the key node of the network and play a pivotal role in the network. Quercetin had the most significant number of targets, with 222 potential targets, followed by luteolin, baicalein, and dinatin, with 74, 63, and 40 potential targets, respectively. These active ingredients with more targets may be the core ingredients of HS.

### 3.5. PPI Network Diagram of Intersecting Targets

**Figure 4:**
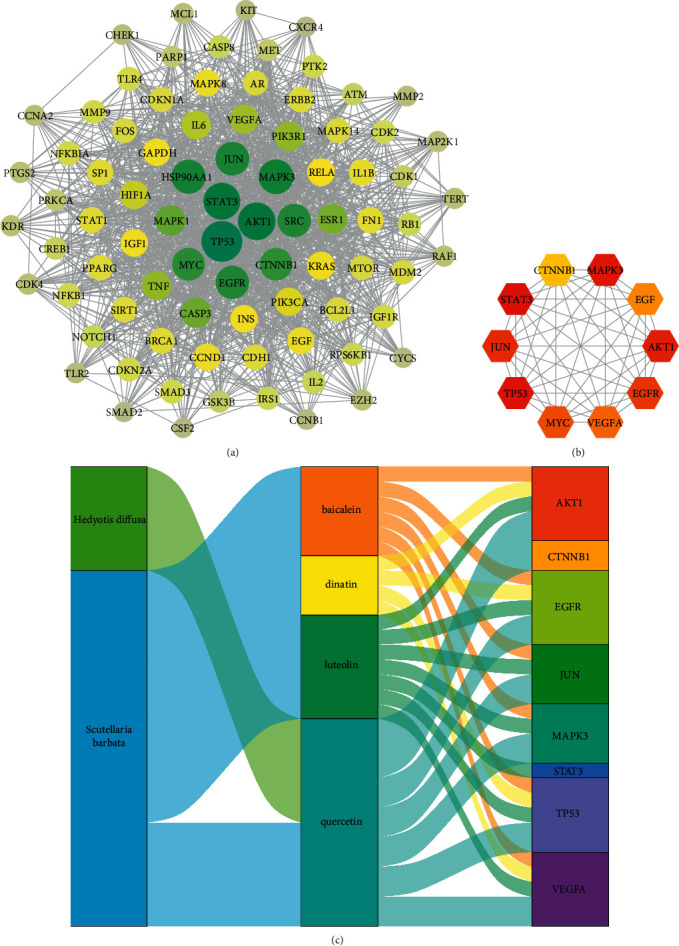
(a) The PPI network of the intersecting targets. (b) The 10 hub genes obtained from the PPI network. (c) The Sankey diagram revealed the relationship between herbs, core ingredients, and hub genes.

The 170 intersecting targets obtained above were imported into the STRING 11.0 database for analysis, and the PPI network diagram of intersection targets between HS and CRC was obtained. There were 166 nodes and 1928 edges in the network, and the characteristics of the specific network topology were calculated. The nodes represent the intersection targets, and the edges represent the association between the intersection targets. We used Cytoscape 3.8.0 to show the PPI network diagram and hide the nodes whose degree value is less than 18.5 (median degree value) (Figure 4(a)). The cytohubba plug-in was used to calculate the targets set to screen the core targets further. The top 10 hub genes were STAT3, TP53, MAPK3, AKT1, JUN, EGFR, MYC, VEGFA, EGF, and CTNNB1 (Figure 4(b)). Furthermore, we revealed the relationship between two herbs, four core ingredients, and ten hub genes using Sankey diagram (Figure 4(c)).

### 3.6. Go and KEGG Analysis of Intersecting Targets

**Figure 5:**
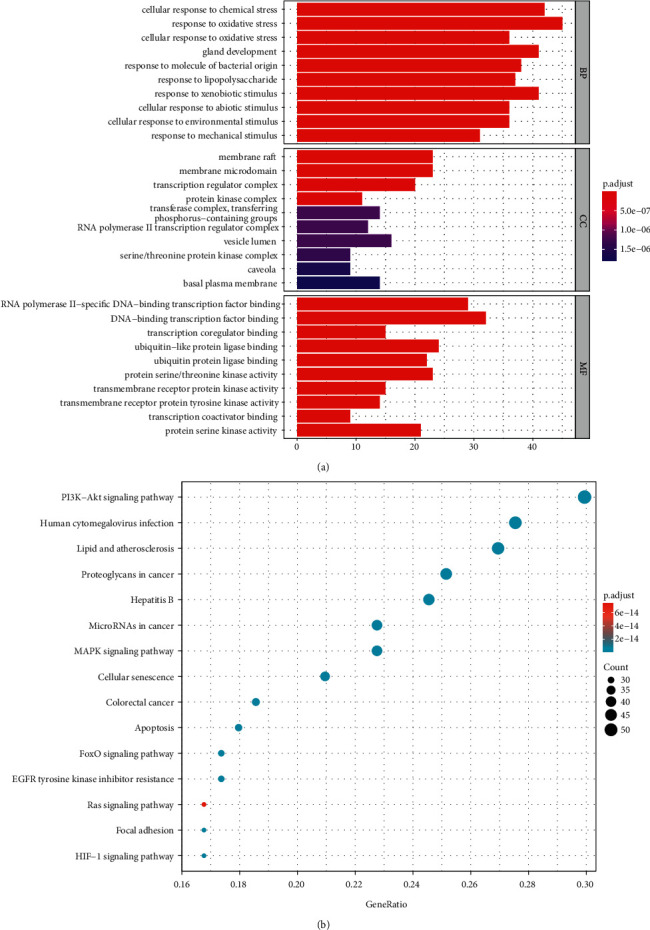
(a) GO enrichment analysis results. (b) KEGG pathway enrichment analysis results.

Based on GO enrichment and KEGG analysis, we determined how these intersecting targets function biologically. As a result, the bar plot of the top 10 terms of biological process (BP), cellular component (CC), and molecular function (MF) terms were displayed (Figure 5(a)). In the three categories, changes in the BP of targets were enriched in cellular response to chemical stress and response to oxidative stress; changes in MF were mainly enriched in transcription factor binding, transcription coregulator binding, and ubiquitin-like protein ligase binding, while CC were mainly enriched in membrane raft, membrane microdomain, and transcription regulator complex. Through enrichment and screening of the KEGG pathway, 168 signaling pathways were obtained. Combined with literature research, we screened out the pathways directly related to CRC and showed the top 15 pathways in the dot plot according to the count value. Among them, the PI3K-Akt signaling pathway, proteoglycans in cancer, and MAPK signaling pathway are closely related to CRC, which may be the critical pathways of HS in the treatment of CRC (Figure 5(b)).

### 3.7. Molecular Docking

**Figure 6:**
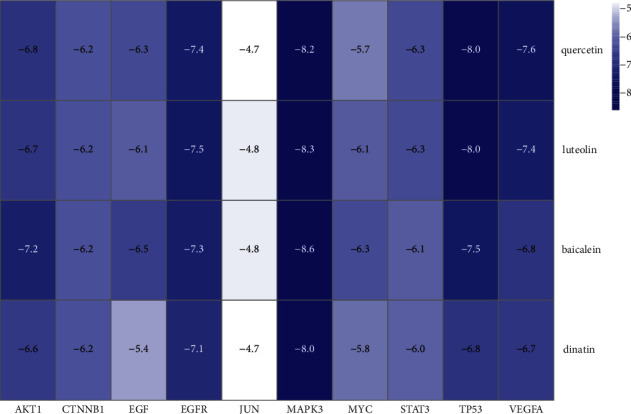
Heatmap of the molecular docking efficiency.

In this study, we conducted molecular docking of 10 hub genes (STAT3, TP53, MAPK3, AKT1, JUN, EGFR, MYC, VEGFA, EGF, and CTNNB1) with four core ingredients (quercetin, luteolin, baicalein, and dinatin) to evaluate the protein-ligand binding potential. The results showed that the four ingredients had the best docking effect with MAPK, and the binding energies were -8.2 kcal/mol, -8.3 kcal/mol, -8.6 kcal/mol, and -8.0 kcal/mol, respectively (Figure 6).

**Figure 7:**
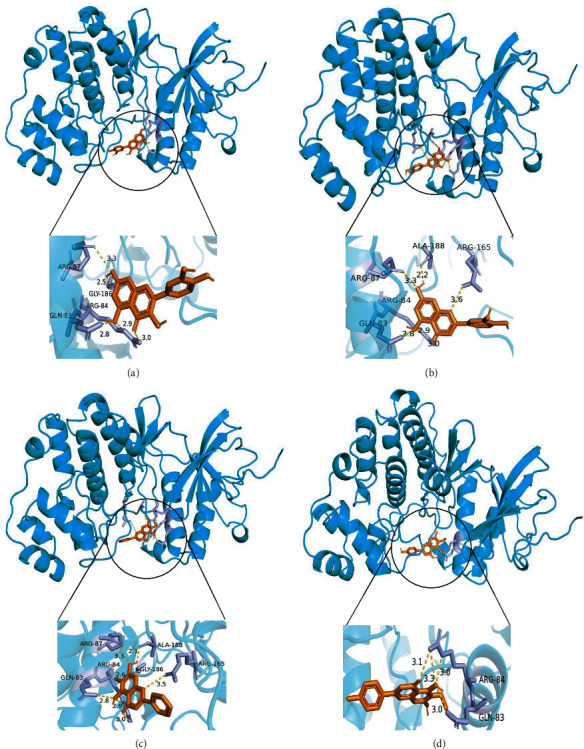
3D diagram of molecular docking models, MAPK3 binds to quercetin (a), luteolin (b), baicalein (c), and dinatin (d).

**Figure 8:**
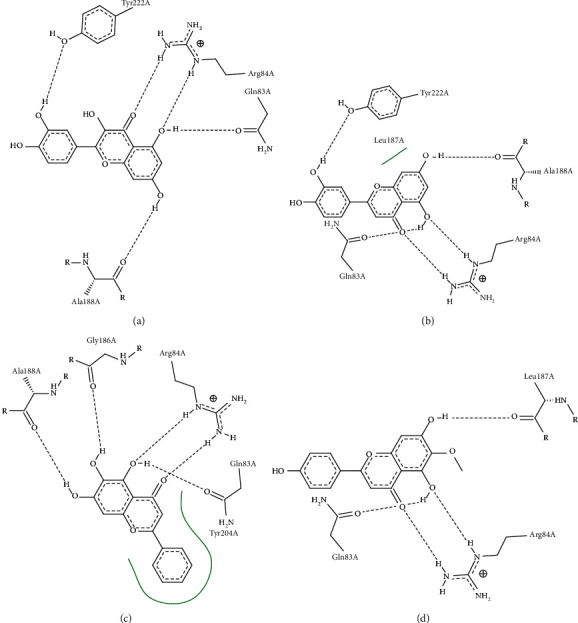
2D diagram of molecular docking models shows the details of the interaction, MAPK3 binds to quercetin (a), luteolin (b), baicalein (c), and dinatin (d).

Quercetin and luteolin also achieved quite good docking results with TP53, and the docking results were -8.0 kcal/mol and -8.0 kcal/mol, respectively. The 3D diagrams of molecular docking models were displayed by PyMol 2.4.0, which showed the interaction of MAPK with quercetin, luteolin, baicalein, and dinatin (Figure 7). At the same time, the 2D diagrams showed the details of the interaction by using Proteins Plus (Figure 8).

## 4. Discussion

Both *Hedyotis diffusa* and *Scutellaria barbata* are heat-clearing and detoxicating TCMs. *Hedyotis diffusa* can also activate the blood circulation and relieve pain, while *Scutellaria barbata* can remove blood stasis and diuresis [38]. They could play a coordinated and synergistic role in treating tumors, and the combined use of the two drugs is not simply superimposed, and their combined application is more effective than a single drug [39]. The antitumor mechanism of *Hedyotis diffusa* is mainly achieved by enhancing immune function, interfering with the energy metabolism of tumor cells, inducing tumor cell apoptosis, and influencing the mitochondrial pathway [40, 41]. However, the antitumor mechanisms of *Scutellaria barbata* include inhibiting tumor cell growth, inducing tumor cell apoptosis, inhibiting tumor angiogenesis and metastasis, regulating immunity, and reversing drug resistance of tumor cells [42, 43].

In other words, some of their functions are the same, especially in inducing apoptosis, enhancing cell immunity, and reducing telomerase activity [40]. They also have their own unique functions, which makes them mutually beneficial to play a more significant role. Some studies have shown that in the breast cancer model mice, the HS group can significantly increase the tumor inhibition rate, serum INF–*γ* and IL-2 levels, and decrease serum TNF-*α* level [16]. It is reported that when the sample concentration was in the medium concentration range (0.5–1.2 mg/ml), HS had a strong chelating ability to Fe^2+^, followed by *Hedyotis diffusa*, and the extract of *Scutellaria barbata* was relatively weak. It is suggested that the herb pair may inhibit or resist tumor formation by chelating the transition metal ions and blocking the lipid peroxidation chain reaction. In addition, HS can provide the most substantial DNA protection, which may be why it works [44].

In this study, the network pharmacology method was used to analyze the active ingredients, targets, and potential mechanisms of HS in CRC treatment. Through the construction of an herb-ingredient-target-disease network and a PPI network of intersecting targets, the mechanism of action of HS on CRC was systematically analyzed.

In the herb-ingredient-target-disease network, quercetin, luteolin, baicalein, and dinatin have more targets than other ingredients, which may be the core active ingredients of HS. Quercetin is one of the main ingredients of *Hedyotis diffusa*, and the other three are the main ingredients of *Scutellaria barbata*. It has been reported that quercetin can not only directly inhibit the proliferation of tumor cells [45, 46], but also plays an antitumor effect by antioxidating [47] and activating antitumor immunity [48] and inhibiting EMT [49, 50]. Luteolin is a natural flavonoid, which can increase ceramide, leading to the apoptosis and death of CRC cells and inhibit the synthesis and metabolism of ceramide complex sphingolipid [51, 52]. In addition, it can inhibit the production of MMP-9 and MMP-2 and up-regulate the expression of TIMP-2, thus playing an antimetastasis role [53–55]. Baicalein is considered to possess antitumor activity, which can effectively disrupt the proliferation, migration, and invasion of CRC cells and decrease the expression of epithelial-mesenchymal transition promoting factors including vimentin, Twist1, and Snail [56, 57]. It also inhibits the proliferation and invasion of human CRC cell lines by reducing the expression of MMP-2 and MMP-9 via regulation of the AKT signaling pathway [58, 59]. Dinatin is also known as hispidulin, and it can induce ROS-mediated apoptosis of human non-small cell lung cancer cells by activating the ER stress pathway and ER stress-induced apoptosis of human liver cancer cells by activating the AMPK/mTOR signaling pathway [60–62]. Moreover, hispidulin treatment significantly inhibited the activity of sphingosine kinase one and consequently promoted ceramide accumulation, thus leading to apoptosis of renal cell carcinoma [63].

In the PPI network，we identified ten hub genes, namely, STAT3, TP53, MAPK3, AKT1, JUN, EGFR, MYC, VEGFA, EGF, and CTNNB1, which may be the targets of HS in the treatment of CRC. The activation of STAT3 could be observed in various tumors, which was closely related to inflammation and immunity and promoted tumor progression as an oncogene [64]. The expression of pSTAT3 increased significantly in tumor-associated fibroblasts and activated angiogenesis-related transcription factors to promote the advancement of CRC [65]. The missense mutation of TP53 (mutp53) was common in CRC, and it was estimated that this mutation existed in more than half of the CRC. Mutp53 limited the binding of SHP and STAT3 and derived cancer growth and invasion by activating STAT3 [66]. MAPK3, also known as ERK1, is a serine/threonine kinase. It was found that the levels of phosphorylated STAT3 and ERK1/2 decreased significantly in 8-gingerol treated CRC cells, resulting in the reduced expression of the downstream target gene c-Myc [67]. The expression of VEGF was regulated by Gab2 and stimulated its downstream genes ERK1/2 and c-Myc in CRC cells [68]. EGFR is a receptor tyrosine kinase, which can act as a regulator of tumor immune monitoring, activate the JAK/STAT3 signaling pathway, and promote the expression of PD-L1 [69]. Overexpression of EGFR and its downstream Ras/Raf/MEK/ERK signaling plays an essential role in regulating cell cycle progression, cell proliferation, and apoptosis [70, 71]. Studies have shown that after EGF treatment, pY291 Fas promoted the nuclear localization of phosphorylated EGFR and phosphorylated STAT3, the expression of cyclin D1, the activation of Akt and MAPK pathways mediated by STAT3 [72]. Transcription factor c-Jun was regulated by phosphatidic acid (a key intermediate of lipid metabolism) and can enhance the transcription of the WEE1 gene, a checkpoint regulator of the cell cycle [73]. The abnormal expression of CTNNB1 was closely related to the progression and metastasis of CRC. Studies have shown that TRAF6 inhibited EMT and CRC metastasis by driving the degradation mechanism of CTNNB1 [74]. The molecular docking results showed that these hub genes and core ingredients have good binding potential, suggesting that HS played a role in the treatment of CRC mainly through these hub genes.

In the pathway enrichment, the PI3K-Akt signaling pathway (hsa04151) has been identified as the critical target of tumor-targeted therapy, which plays a vital role in regulating the proliferation, migration, and apoptosis of tumor cells [75–77]. Phosphoinositide 3-kinase was a member of the intracellular lipid kinases and regulated cell proliferation and differentiation [78]. AKT activation drove both glycolytic metabolism of glucose and mitochondrial metabolism that generated acetyl-CoA, the biosynthetic precursor of fatty acids, cholesterol, and isoprenoid synthesis. Akt signal transduction could activate the mTORC1 complex, and mTORC1 stimulated adipogenesis by regulating SREBP-mediated FASN expression [79]. The PI3K/Akt/mTOR pathway inhibitors provided a promising target for the treatment of CRC. Proteoglycans in the cancer pathway (hsa05205) revealed the role of proteoglycan in the growth, metastasis, and dissemination of cancer cells. For example, biglycan, as a proteoglycans, combined with vascular endothelial growth factor, can promote the progression of CRC by inducing the increase of vascular density [80]. MAPK signaling pathway (hsa04010) could also be associated. MAPK axis was downstream of many membrane receptors, including EGFR, which transmits extracellular signals to the nucleus and regulates various cellular functions [81]. The RAS/MAPK signaling pathway was an essential pathway in the proliferation, differentiation, and invasion of CRC, as activated RAS triggers the activation of RAF and subsequently activated RAF phosphorylates activates MEK, which phosphorylates and activates MAPK/ERK [82]. In addition, we found that colorectal cancer (hsa05210) was directly related to CRC and was an essential regulator of cell proliferation, apoptosis, and genomic stability [15].

## 5. Conclusion

In this study, we screened four core ingredients (quercetin, luteolin, baicalein, and dinatin) from 33 HS active ingredients and obtained 170 intersection targets related to CRC. The top 10 hub genes were STAT3, TP53, MAPK3, AKT1, JUN, EGFR, MYC, VEGFA, EGF, and CTNNB1, which may be the core targets of HS. Our study shows that HS acts simultaneously on a variety of signaling pathways related to CRC, such as the PI3K-Akt signaling pathway, proteoglycans in cancer, and the MAPK signaling pathway, which provides a reference for the research on the specific mechanism of the drug in the treatment of CRC. However, the limitation of this study is that network pharmacology ignores the content of each ingredient in the drug. In addition, the current research methods ignore the possible production of new compounds in the process of drug decoction. At the same time, another limitation is that network pharmacology has a high false-positive rate. It is necessary to illustrate the molecular level of HS on the treatment of CRC in the future. However, this screening technology, combined with network pharmacology and molecular docking, saves a lot of scientific research resources and helps researchers screen drug ingredients and core targets efficiently. In the follow-up research, on the one hand, we could continue to study the effect of HS on the treatment of CRC at the molecular level. On the other hand, we need to constantly optimize the technology related to drug screening to achieve more accurate drug screening and reduce the false-positive rate. We hope that our research can bring a new perspective to the therapy of CRC and find new anticancer drugs.

## Figures and Tables

**Table 1:** Basic information of 33 active ingredients in HS.

Mol ID	Molecule name	OB (%)	DL	Targets	Herb
MOL000098	Quercetin	46.43	0.28	369	H/S
MOL000358	Beta-sitosterol	36.91	0.75	49	H/S
MOL000449	Stigmasterol	43.83	0.76	42	H/S
MOL001646	2,3-Dimethoxy-6-methyanthraquinone	34.86	0.26	0	H
MOL001659	poriferasterol	43.83	0.76	42	H
MOL001663	(4aS,6aR,6aS,6bR,8aR,10R,12aR,14bS)-10-Hydroxy-2,2,6a,6 b,9,9,12a-heptamethyl-1,3,4,5,6,6a,7,8,8a,10,11,12,13,14b-tetradecahydropicene-4a-carboxylic acid	32.03	0.76	84	H
MOL001670	2-Methoxy-3-methyl-9,10-anthraquinone	37.83	0.21	100	H
MOL000006	Luteolin	36.16	0.25	216	S
MOL000173	Wogonin	30.68	0.23	53	S
MOL000351	Rhamnazin	47.14	0.34	100	S
MOL000359	Sitosterol	36.91	0.75	49	S
MOL000953	CLR	37.87	0.68	0	S
MOL001040	(2R)-5,7-Dihydroxy-2-(4-hydroxyphenyl) chroman-4-one	42.36	0.21	83	S
MOL001735	Dinatin	30.97	0.27	120	S
MOL001755	24-Ethylcholest-4-en-3-one	36.08	0.76	46	S
MOL001973	Sitosteryl acetate	40.39	0.85	23	S
MOL002714	Baicalein	33.52	0.21	180	S
MOL002719	6-Hydroxynaringenin	33.23	0.24	15	S
MOL002776	Baicalin	40.12	0.75	51	S
MOL002915	Salvigenin	49.07	0.33	100	S
MOL005190	Eriodictyol	71.79	0.24	22	S
MOL005869	Daucostero_qt	36.91	0.75	0	S
MOL008206	Moslosooflavone	44.09	0.25	100	S
MOL012245	5,7,4′-Trihydroxy-6-methoxyflavanone	36.63	0.27	70	S
MOL012246	5,7,4′-Trihydroxy-8-methoxyflavanone	74.24	0.26	88	S
MOL012248	5-Hydroxy-7,8-dimethoxy-2-(4-methoxyphenyl) chromone	65.82	0.33	0	S
MOL012250	7-Hydroxy-5,8-dimethoxy-2-phenyl-chromone	43.72	0.25	0	S
MOL012251	Chrysin-5-methylether	37.27	0.2	100	S
MOL012252	9,19-Cyclolanost-24-en-3-ol	38.69	0.78	26	S
MOL012254	Campesterol	37.58	0.71	15	S
MOL012266	Rivularin	37.94	0.37	11	S
MOL012269	Stigmasta-5,22-dien-3-ol-acetate	46.44	0.86	26	S
MOL012270	Stigmastan-3,5,22-triene	45.03	0.71	0	S

## Data Availability

The data used in the study are available upon request to the corresponding author.
